# Synthetic biology toolkit of *Ralstonia eutropha (Cupriavidus necator)*

**DOI:** 10.1007/s00253-024-13284-2

**Published:** 2024-08-29

**Authors:** Lara Santolin, Sebastian L. Riedel, Christopher J. Brigham

**Affiliations:** 1 Technische Universität Berlin, Institute of Biotechnology, Chair of Bioprocess Engineering, Berlin, Germany; 2 Berliner Hochschule Für Technik, Department VIII – Mechanical Engineering, Event Technology and Process Engineering, Environmental and Bioprocess Engineering Laboratory, Berlin, Germany; 3 Department of Bioengineering, University of Massachusetts Dartmouth, North Dartmouth, MA USA

**Keywords:** *Ralstonia eutropha* (*Cupriavidus necator*), Synthetic biology toolkit, Standardized parts, Metabolic engineering, Bioproduction, Industrial biotechnology

## Abstract

**Abstract:**

Synthetic biology encompasses many kinds of ideas and techniques with the common theme of creating something novel. The industrially relevant microorganism, *Ralstonia eutropha* (also known as *Cupriavidus necator*), has long been a subject of metabolic engineering efforts to either enhance a product it naturally makes (polyhydroxyalkanoate) or produce novel bioproducts (e.g., biofuels and other small molecule compounds). Given the metabolic versatility of *R. eutropha* and the existence of multiple molecular genetic tools and techniques for the organism, development of a synthetic biology toolkit is underway. This toolkit will allow for novel, user-friendly design that can impart new capabilities to *R. eutropha* strains to be used for novel application. This article reviews the different synthetic biology techniques currently available for modifying and enhancing bioproduction in *R. eutropha*.

**Key points:**

• *R. eutropha (C. necator) is a versatile organism that has been examined for many applications*.

• *Synthetic biology is being used to design more powerful strains for bioproduction*.

• *A diverse synthetic biology toolkit is being developed to enhance R. eutropha’s capabilities*.

## Introduction

The term “synthetic biology” has existed since before the early 1980s, referring to genetically engineered bacteria designed to perform a specific task (Benner & Sismour 2005). Since then, the term has acquired a variety of aspects and taken on several different, though not necessarily mutually exclusive, definitions. At the turn of the new century, the term “synthetic biology” was used to describe novel (read: “unnatural”) organic molecules functioning in living cells (Rawls 2000). Indeed, these days, the term encompasses both of the aforementioned endeavors, and many additional types of study, including metabolic modelling, specific recombinant DNA assembly processes, and development of cells with stripped-down genomes. In short, the emerging discipline of synthetic biology is built on a multidisciplinary foundation and encompasses a wide range of subdisciplines.

Synthetic biology can generally be thought of as engineering biology. To wit, synthetic biology even has its own multi-step, cyclic design process (the Design-Build-Test (DBT) cycle) similar to the engineering design process (Baldwin et al. 2016; National Academies of Sciences, Engineering and Medicine 2018). Semi-automated, iterative metabolic engineering is even possible by implementing machine learning methods that are able to recommend new strain designs for the next DBT cycle (van Lent et al. 2023). In most classical engineering disciplines, problems are solved, and products are made with the aid of standardized parts. The concept of standardization is a relatively new one in biology. Standards can improve the synthetic biology design process by providing better communication between workers and groups, interchangeability, reproducibility, quality assurance, safety, etc. (Müller & Arndt 2012). Standardization comes in part from published documents called standards, and these documents are published by a variety of professional societies and other entities. Consumer products can be repeatably and reliably manufactured thanks to the existence of standardized parts (e.g., bolt and screw threads). The biosciences have had standards in the form of synthetic growth media formulations, chemical compositions, and other aspects of manufacturing products for biological research (Benner & Sismour 2005). However, only recently have there existed standardized DNA parts for molecular biology and construction of novel strains and cell lines in bioengineering. Leading the way in developing standardized parts for assembly of genetic devices and systems are organizations like BioBricks Foundation (biobricks.org) and the International Genetically Engineered Machine foundation (iGEM, www.igem.org). Using these standardized parts, the goal is to be able to reliably construct genetic devices that impart novel and useful functions to the host cell.

One of the targets of synthetic biology is to take specific genetic parts and assemble them to synthesize a genetic device. That novel genetic device would then be active when introduced to the proper host organism. Many different types of organisms can be used and in fact have been used as host organisms (i.e., microbial chassis). There are a number of aspects that make a good synthetic biology chassis organism, including the ability to survive environmental conditions of the desired application, metabolic versatility, and the existence of reliable molecular genetic tools for use in the organism (Kim et al. 2016b). Common chassis organisms like *Escherichia coli*, *Bacillus subtilis*, and *Saccharomyces cerevisiae* all possess the qualities of a suitable synthetic biology host. Another microorganism that, based on numerous genetic, metabolic, and physiological studies, presents itself as a promising synthetic biology chassis is the Gram-negative non-pathogenic betaproteobacterium *Ralstonia eutropha* (also known as *Cupriavidus necator*).

The wild-type *R. eutropha* H16 is an integral biocatalysis chassis for the production of high-value compounds including polyhydroxyalkanoate (PHA) biodegradable bioplastics (Chen 2009; Morlino et al. 2023), alcohol biofuels (Panich et al. 2021), fine chemicals (Hanko et al. 2022; Lu et al. 2016; Milker & Holtmann 2021), and enzymes (Srinivasan et al. 2002). This versatile, facultative chemolithotroph naturally utilizes a wide range of renewable raw and waste organic carbon sources and alternatively fixes CO_2_ using H_2_ as an energy source under aerobic growth conditions (Lenz et al. 2010), can be cultured to very high cell densities > 200 g L^−1^ (Ryu et al. 1997), and has an extensive synthetic biology toolkit available (Pan et al. 2021) making it the ideal host strain for the sustainable production of valuable products via synthetic biology-driven strain engineering.

As a model organism for PHA production, *R. eutropha* naturally accumulates up to 90 % of its cell dry weight as intracellular PHA granules (Jendrossek & Pfeiffer 2014; Reinecke & Steinbüchel 2008). The polymer, which serves the bacterium as a carbon and energy reservoir and increases its robustness against various stresses (Obruca et al. 2020), can be biotechnologically exploited as a green plastic substitute. Depending on the monomer content of the polymer, PHA exhibits tailorable material properties mimicking the best-selling petrochemical plastics (Doi et al. 1995; Thiele et al. 2024a, b) and is completely biodegradable in common natural environments, leaving no microplastic traces (Laycock et al. 2020). Also, this microbial biopolymer is biocompatible, showing great potential for the design of biomedical devices (Gregory et al. 2022). With an installed PHA manufacturing capacity in 2023 estimated at 105 kt/annum (European Bioplastics, nova-Institute 2023), PHAs have potential applications in packaging and biomedicine and as non-woven fabrics, among others (Mahato et al. 2023).

The favorable growth capabilities of *R. eutropha* extend across autotrophic, mixotrophic, and heterotrophic conditions. During heterotrophic growth, the organism efficiently utilizes sugars, predominantly fructose, N-acetylglucosamine, and gluconate, via the Entner–Doudoroff pathway (Volodina et al. 2016). It also exhibits excellent growth on feedstocks containing diverse fatty acids, including plant oils and waste animal fats (Riedel & Brigham 2020), facilitated by the natural secretion of lipases that mediate the hydrolysis of the triacylglycerols forming natural emulsions (Gutschmann et al. 2021). Further, *R. eutropha* utilizes glycerol and was also reported to grow on some aromatic compounds (Fukui et al. 2014; Johnson & Stanier 1971). When organic substrates are not readily available, the bacterium can grow on CO_2_ or formate, driving autotrophic CO_2_ fixation via the Calvin–Benson–Bassham (CBB) cycle while three distinct oxygen-tolerant [NiFe]-hydrogenases deliver energy via H_2_ oxidation with O_2_ serving as the final electron acceptor (Borrero-de Acuña & Poblete-Castro 2023; Burgdorf et al. 2006; Lenz et al. 2010). In the absence of oxygen, *R. eutropha* can switch to anaerobic denitrification, using nitrite (NO_2_^−^) or nitrate (NO_3_^−^) as alternative electron acceptors (Pfitzner & Schlegel 1973).

The sequencing of the complete genome of *R. eutropha* (Pohlmann et al. 2006), along with comprehensive studies in transcriptomics, proteomics, and metabolomics (Morlino et al. 2023), provides invaluable insights into the metabolic pathways of the organism. These advancements, complemented by the vast improvement of the available synthetic biology toolkit (Pan et al. 2021), including the recent application of CRISPR/Cas (Wang et al. 2024c; Xiong et al. 2018), allow for rational engineering of production strains, increasing versatility towards utilizing low-cost feedstocks and diversifying the product portfolio of the organism. Altogether, synthetic biology techniques specially developed for *R. eutropha* present a landscape of endless possibilities for the future exploration and exploitation of this remarkable microbial chassis.

## Synthetic biology of *R. eutropha*

**Fig. 1:**
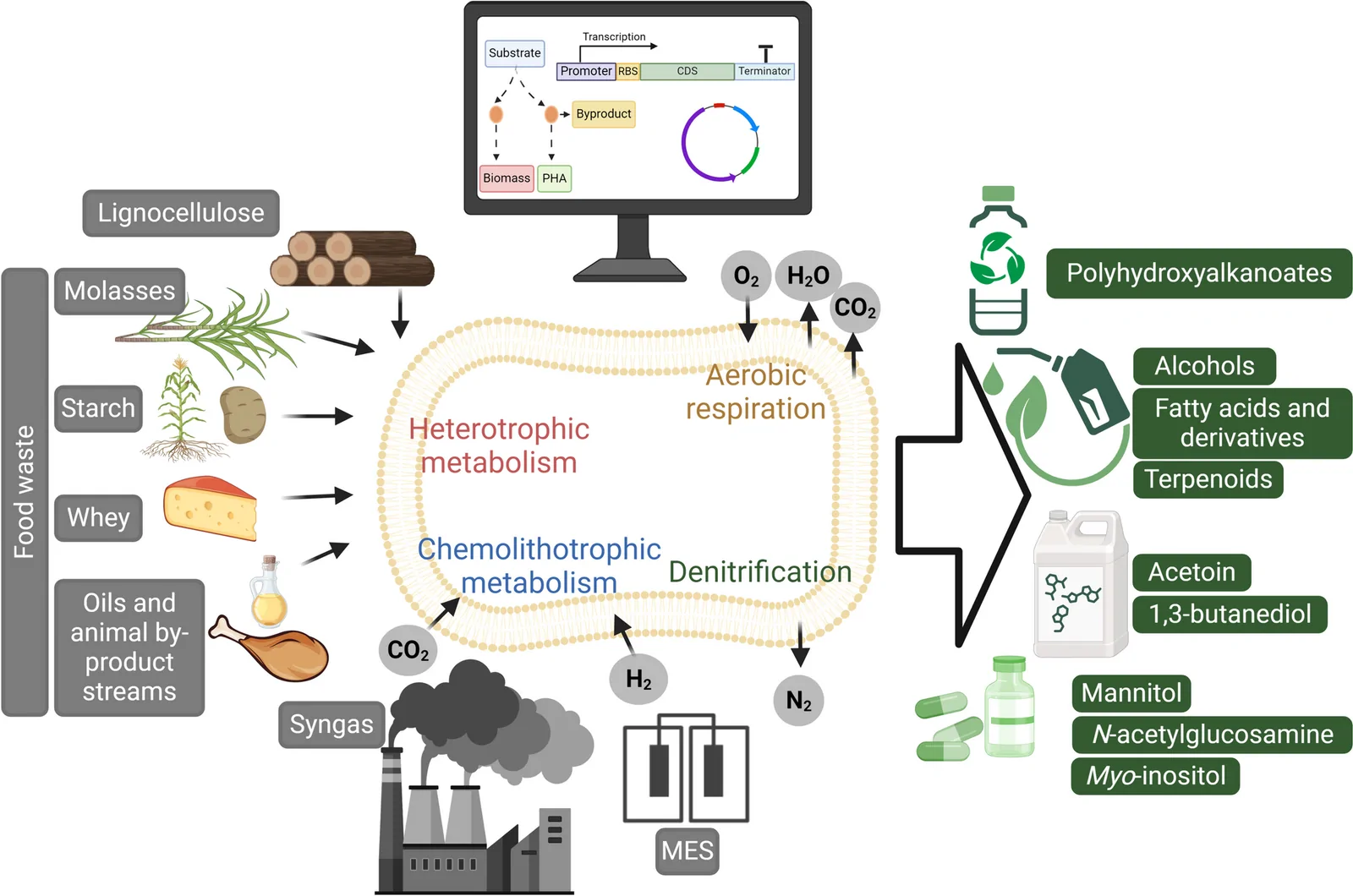
Engineering of *R. eutropha* as an integral production platform of bioplastics, biofuels, platform chemicals, and dietary supplements

To expand the substrate utilization range and product portfolio of *R. eutropha*, and as a result establish it as an integral biocatalysis platform aligned with the requirements of a biorefinery concept (Fernando et al. 2006), precise expression systems and efficient genome editing tools are key to realize the integration and control of heterologous genes. At the same time, a deep understanding of the metabolic pathways of *R. eutropha*, along with the design and implementation of metabolic models, is essential to identify metabolic bottlenecks and develop efficient biotechnological processes (Fig. 1).

### Developing promoters for expression and repression systems

Precise control of gene expression is key to the development of efficient production strains. Numerous genetic engineering vectors, including episomal plasmids with robust stability, as well as integrative plasmids for chromosomal insertion, have been developed for *R. eutropha* by several groups, and these efforts have been the subject of a recent review (Pan et al. 2021).

When designing genetically engineered strains, promoters are key elements determining transcription efficiency, leading to the optimization of gene expression. A vast library of constitutive promoters, including native promoters (Brigham et al. 2012; Gruber et al. 2014; Hein & Steinbüchel 1996; Li & Liao 2015; Priefert & Steinbuchel 1992), *E. coli*-derived promoters (Arikawa & Matsumoto 2016; Fukui et al. 2011), T4 and T5 bacteriophage-derived promoters (Gruber et al. 2014), and also synthetic promoters (Alagesan et al. 2018; Johnson et al. 2018), is currently available. Nevertheless, comparing the relative strength of these promoters (Table 1) is challenging as there is no standardized characterization strategy. For this, some groups have used β-galactosidase reporter gene assays, while others have measured the expression of some fluorescent protein. Furthermore, slight differences in promoter architecture and cultivation conditions are always found between different studies. Overall, *R. eutropha* native promoters rank among the weakest of those examined, with the PHA synthase promoter P_*phaC1*_ being the most used. Higher expression levels can be achieved with heterologous constitutive promoters derived from *E. coli*, including the *lac* promoter (P_*lac*_) and the P_*tac*_ derivation that in *R. eutropha* are not inducible with IPTG due to the absence of *lacI* and *lacY* homologues (Arikawa & Matsumoto 2016; Fukui et al. 2011). High gene expression is also observed in *R. eutropha* using bacteriophage T4- and T5-derived promoters. To date, the highest reporter gene expression was achieved from P_22_, a synthetic promoter developed by Alagesan et al. (2018) which was constructed by recombineering core promoter sequences and upstream and downstream insulation sequences of previously characterized promoters from *R. eutropha*, *E. coli*, and bacteriophages T4 and T5 (Alagesan et al. 2018).

**Table 1:** Constitutive promoters used in *R. eutropha*

Promoter	Origin	Relative strength [RFU]
P_*groEL*_	Native, heat shock chaperone	600^1^
P_*rrsC*_	Native, translation	900^2^
P_*phaC1*_	Native, PHA synthase promoter	1000^3^
P_*pdhE*_	Native, pyruvate metabolism	1300^2^
P_*B1772*_	Native, uncharacterized	3100^1^
P_*lac*_	*E. coli*, lactose metabolism	3500^1^
P_*tac*_	*E. coli*, synthetic (combination of P_lac_ and P_trp_)	4300^1^
P_*trp*_	*E. coli*, tryptophan synthesis	4400^4^
P_*lacUV5*_	*E. coli*, mutated P_lac_	9300^4^
P_*trc*_	*E. coli*, synthetic (combination of P_lac_ and P_trp_)	10,000^4^
P_*j5*_	T5 bacteriophage	20,000^1^
P_22_	Synthetic	65,000^5^

^1^(Gruber et al. 2014); ^2^β-galactosidase reporter assay values were taken from (Black et al. 2018) and normalized to the relative strength of P_*phaC1*_; ^3^(Fukui et al. 2011); ^4^β-galactosidase values were taken from Arikawa & Matsumoto (2016) and normalized to the relative strength of P_*phaC1*_; ^5^values were taken from Alagesan et al. (2018) and correlated with ^1^

In order to minimize metabolic burden, enhance product yield, and prevent potential toxic effects, inducible gene expression systems are mostly preferred. In *R. eutropha*, some native, auto-inducible systems, which avoid additional process costs related to the implementation of a potentially costly inducer molecule, are available, including P_*phaP*_ activated under PHA-permissive conditions by release of PhaR (Mitra et al. 2022; Santolin et al. 2024), P_*cbbL*_ induced under autotrophic conditions (Raberg et al. 2018), P_*acoD*_ and P_*acoX*_ induced by acetoin (Delamarre & Batt 2006), and the hydrogenase promoters P_*SH*_ and P_*MBH*_, the strongest among all with up to ninefold induction factor, induced by glycerol (Jugder et al. 2016; Schwartz et al. 1999). However, ultimately heterologous, inducible systems that show orthogonality to *R. eutropha* are applied as they enable a more precise expression control, independent of the metabolic state of the cells, and offer higher induction ratios. Among these, the carbon catabolite repression-based, L-arabinose-inducible AraC/P_*araBAD*_ system is broadly employed showing high transcription levels, although high concentrations (1 g L^−1^) of inducer are necessary to achieve reasonable, long-term induction, and inhibition of cell growth by this inducer has been observed (Alagesan et al. 2018; Bi et al. 2013; Fukui et al. 2011; Hanko et al. 2022). Although with lower overall transcription levels, the RhaRS/P_*rhaBAD*_-L-rhamnose system can enable fine-tuning of gene expression in *R. eutropha* showing the highest induction factor reported to date (1960-fold) (Alagesan et al. 2018). Synthetic systems have also been developed by combining different regulatory units in an effort to achieve high induction efficiencies. In this context, an anhydrotetracycline-inducible system was developed by fusing the *tetO* operator to the P_*ccrC*_ promoter and tuning synthesis of the repressor TetR by a P_*phaC1*_ expression library which resulted in an induction factor around 1100-fold (Li & Liao 2015). Nevertheless, the expensive inducer makes the system economically unfeasible for large-scale applications. The *tetO*/TerR system was further combined with the doxycycline inducible P_*tolC*_ promoter in an effort to reduce costs linked to the inducer, although a far lower induction ratio was achieved (Aboulnaga et al. 2018). Recently, an optimized T7 expression system for *R. eutropha* was constructed with the T7 RNA polymerase gene driven by the AraC/P_*araBAD*_ promoter and by this the inducer concentration could be decreased 20 times offering great cost saving (Hu et al. 2020). Inducible systems are particularly relevant when the strong expression of a product results in growth inhibition/toxicity. By applying such systems, the production of the potentially toxic compound can be triggered strictly after the growth phase, thereby maximizing product yields. In connection to this, whereas heterologous protein production in *E. coli* often results in the formation of inclusion bodies (IB), the formation of IB is suppressed in *R. eutropha*, yielding soluble and active protein (Bernard et al. 2004).

As discussed above, a number of native, heterologous, and synthetic, constitutive, and inducible expression systems have been developed for use in *R. eutropha.* However, to deal with complex metabolic engineering tasks and promote the reusability of available promoter libraries, modular and standardized cloning systems that enable a combinatorial assembly of promoters, ribosome binding sites (RBS) and terminators should be implemented. In this context, a Type IIS modular cloning assembly method allowing the design of complex genetic circuits containing multiple transcription units guided by a user-friendly and intuitive computational pipeline has been developed and validated for a close relative of *R. eutropha*, *Cupriavidus metallidurans* (Blázquez et al. 2023). It is possible, perhaps even likely, that such a combinatorial assembly system could be used in *R. eutropha* strains, as well.

Regarding programmable gene repression, to date, only one recent study developed a robust strategy for repressing gene expression in *R. eutropha*. Wang and coworkers designed a CRISPR interference system (CRISPRi) using an engineered dCas9 lacking DNA cleavage capability but retaining DNA binding capacity under the guidance of a single guide RNA, posing a steric hindrance effect that effectively inhibits transcription (Wang et al. 2024a). By this, the authors were able to tailor the expression level of a fluorescence protein reporter as well as the production of polyhydroxybutyrate (PHB). Furthermore, the same study applied CRISPRi to achieve increased mutation rates during adaptive laboratory evolution (ALE) by disturbing the expression of the DNA mismatch repair gene *mutS.* Although presenting great promise for programmable gene repression, the system still has a major drawback in the nonignorable toxicity of the dCas9 protein to *R. eutropha*, thus strongly limiting its application. The authors suggest integrating the CRISPRi system into the chromosome and testing other Cas proteins or using ALE to improve robustness of the strain against metabolic burden as possible solutions to increase the potential of this genetic tool.

### Metabolic engineering of carbon sources

**Fig. 2:**
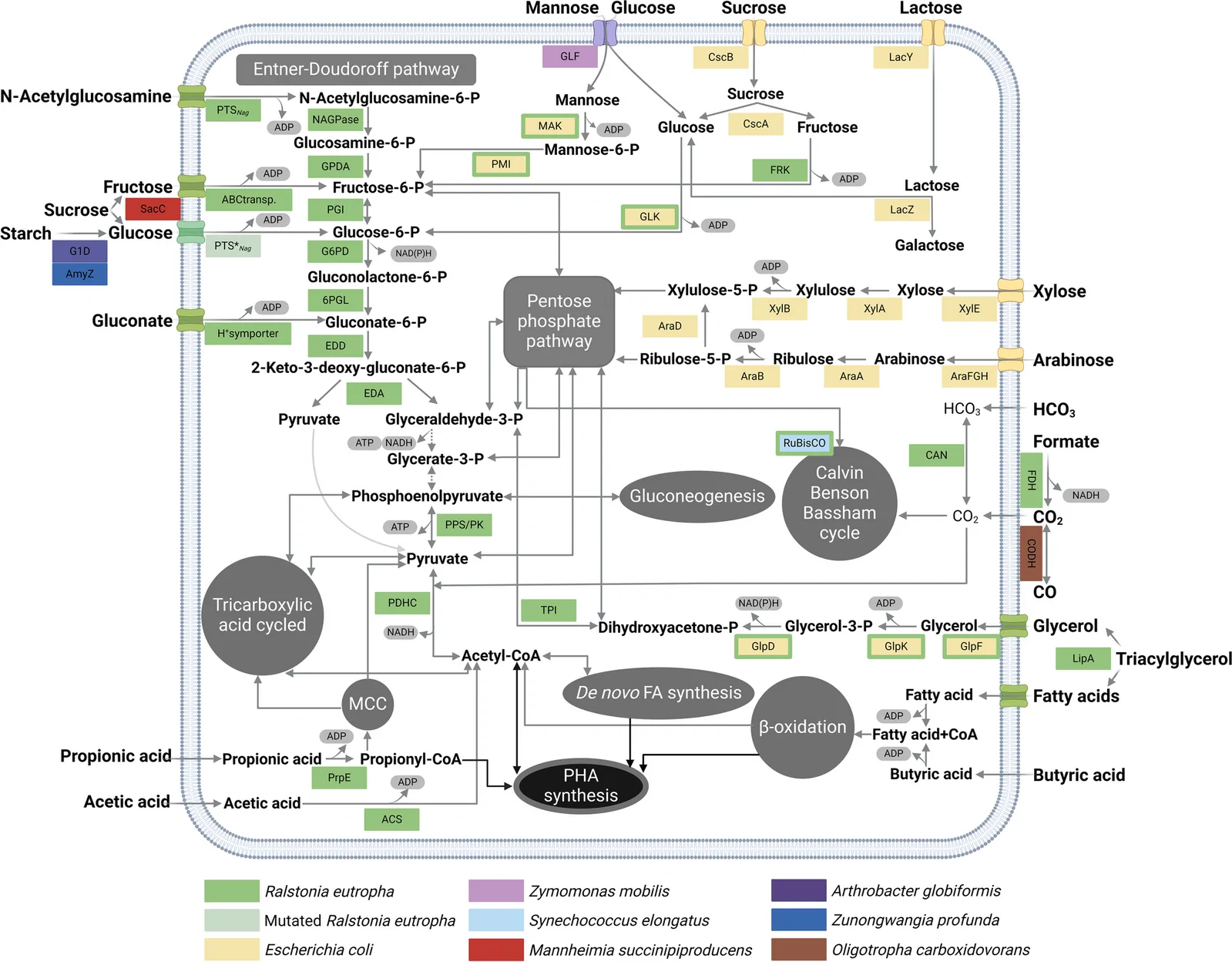
Metabolic engineering of carbon sources in *Ralstonia eutropha*. Abbreviations of enzymes: PTSNag, phosphotransferase system; NAGPase, N-acetylglucosamine-6-phosphate deacetylase; GPDA, glucosamine-6-phosphate deaminase; PGI, phosphoglucose isomerase; G6PD, glucose-6-phosphate dehydrogenase; 6PGL, 6-phosphogluconolactonase; EDD, gluconate-6-phosphate dehydratase; EDA, 2-keto-3-deoxygluconate-6-phosphate aldolase; TPI, triosephosphate isomerase; PK, pyruvate kinase; PPS, phosphoenolpyruvate synthetase; PDHC, pyruvate dehydrogenase complex; GLF, glucose-facilitated diffusion transporter; MAK, mannofructo-hexokinase; PMI, phosphomannose isomerase, GLK, glucokinase; CscB, sucrose permease; CscA, sucrose hydrolase; FRK, fructokinase; LacY, β-galactoside permease; LacZ, β-galactosidase; XylE, xylose-proton symporter; XylA, xylose isomerase; XylB, xylulokinase; AraFGH, arabinose transport system; AraA, arabinose isomerase; AraB, ribulose kinase; AraD, ribulose-5-P-epimerase; CAN, carbonic anhydrases; RuBisCO, ribulosebisphosphate carboxylase; FDH, formate dehydrogenase; CODH, carbon monoxide dehydrogenase; LipA, lipase; GlpF, aquaglyceroporin; GlpK, glycerol dehydrogenase; GlpD, glycerol-3-phosphate dehydrogenase; ACS, acetyl-CoA synthetase; PrpE, propionyl-CoA synthetase. MMC, methylcitrate cycle. Uptake of fatty acids is probably mediated by porins. Volatile fatty acids such as acetic acid, propionic acid, and butyric acid are capable of freely diffusing through the membrane

In the transition towards a circular bioeconomy, the utilization of low-cost feedstocks, including carbon-rich waste streams, is key for the success of biotechnological processes. By this, loops can be established wherein byproducts from industrial processes are fed to microbial cell factories and are then transformed into value-added compounds, which optimizes resource usage, reduces waste generation, and makes the production of these value-added compounds more affordable. In this regard, many synthetic biology efforts have been directed towards conferring *R. eutropha* with the ability to utilize inexpensive raw and waste materials, such as lignocellulosic biomass, molasses, and starch, and further optimizing growth capabilities on carbon sources like glycerol and C1 compounds present in syngas (Fig. 2). The valorization of syngas, consisting mainly of CO_2_ and CO, is particularly relevant because it could allow for mitigation of greenhouse gas (GHG) emissions from industrial activities.

#### Engineering strategies for increased carbohydrate uptake (lignocellulosic biomass, molasses, starch, whey-derived feedstocks)

Sugars stand out as highly favorable, energy-efficient substrates for microbial growth. Unfortunately, wild-type *R. eutropha* H16 can only naturally utilize fructose, N-acetyl-glucosamine, and gluconate as carbon sources, strongly restricting the utilization of waste biomass from food, agriculture, and forest industries, such as lignocellulose, starch, and molasses.

Lignocellulose is the most abundant raw material on earth and is rich in sugars including glucose, mannose, xylose, and arabinose. It is an excellent second-generation feedstock for industrial fermentations and, therefore, engineering *R. eutropha* to utilize this carbon feedstock would confer significant advantages. In *R. eutropha*, the lack of a glucose transport and phosphorylation system prevents glucose uptake and metabolism (Orita et al. 2012). The first glucose-utilizing strain, NCIMB 11599, was obtained by UV mutagenesis (Schlegel & Gottschalk 1965) and showed mutations in the *N*-acetylglucosamine-specific phosphoenolpyruvate-dependent phosphotransferase system (PEP-PTS) that positively affected glucose uptake. An alternative glucose-utilizing strain was developed by heterologous expression of the energy-independent glucose-facilitated diffusion transporter (*glf*) from *Zymomonas mobilis* and co-expression of glucokinase (*glk*) from *E. coli* (Sichwart et al. 2011). Nevertheless, spontaneously evolved glucose-utilizing strains have mostly been applied in literature as they exhibit faster growth rates that are comparable to that of the wild-type strain on fructose. Very recently, Wang and coworkers engineered the complete Embden-Meyerhof Parnas (EMP) pathway into *R. eutropha* by expressing a 6-phosphofructokinase (*pfkA*) from *E. coli*, suggesting that reconstruction of the EMP pathway boosts glucose utilization efficiency in the strain. However, careful consideration of ATP balance is essential when overexpressing *pfkA*, given the higher ATP throughput of the EMP pathway, as compared to the ED pathway (Wang et al. 2023a). Furthermore, *R. eutropha* H16 lacks mannose-specific transporters but harbors a native mannofructo-hexokinase (*mak*) and a phosphomannose isomerase (*pmi*), the expression of which would convert mannose-6-phosphate, to fructose-6-phosphate. Nevertheless, phosphorylation of mannose by the native *mak* was found to be less efficient than phosphorylation of fructose and glucose (Volodina et al. 2016). In order to metabolically favor the utilization of mannose, Sichwart et al*.* expressed *mak* and *pmi* genes from *E. coli* in addition to the *glf* gene, whose product mediates not only the transport of glucose but also of mannose. The resulting engineered strains exhibited moderate growth on mannose as the principal carbon source (Sichwart et al. 2011). Similarly, strategies involving heterologous expression of xylose and arabinose metabolism genes from *E. coli* have been employed to enable *R. eutropha* to utilize these hemicellulose-derived sugars. To engineer xylose utilization, the xylose isomerase pathway can be established by expressing xylose isomerase (*xylA)* and xylulokinase (*xylB)* that catalyze the conversion of intracellular xylose to xylulose-5-phosphate which can enter the non-oxidative pentose phosphate pathway (PPP) present in *R. eutropha* (Kim et al. 2016a). Although growth on the substrate could be demonstrated by this strategy, co-expression of the xylose-proton symporter (*xylE*) conferred a significant improvement in xylose uptake (Liu et al. 2014; Weng et al. 2023). However, utilization of xylose in the engineered strains is inhibited in the presence of glucose, probably through carbon catabolite repression (CCR), which is a relevant throwback as lignocellulose hydrolysates contain both glucose and xylose. Similarly, heterologous expression of the *araBAD* genes in *R. eutropha* permitted utilization of arabinose, and coexpression of *araFGH* improved the uptake of this substrate (Lu et al. 2013). Overall, relatively low growth rates are obtained using xylose and arabinose, which could probably be increased by overexpressing native or heterologous transketolases and transaldolases of the non-oxidative PPP, therefore increasing the metabolic flux to glyceraldehyde-3-P, which is mostly catabolized to acetyl-CoA, thus boosting NADPH cofactor levels. Apart from yielding monosaccharides, the pretreatment of lignocellulosic biomass leads to the degradation of lignin to aromatic compounds like ferulic acid and *p*-coumaric acid and the generation of toxic byproducts like furfural, which can affect microbial growth and productivity. Furfural is generally converted to less toxic compounds through reduction by NAD(P)H-dependant aldehyde reductases. In a recent study, the nicotinamide salvage pathway genes *pncB* and *nadE* were inserted into *R. eutropha* NCIMB 11599 to increase its NAD(P)H pool, thereby enhancing tolerance to lignocellulosic-derived inhibitors (Lee et al. 2022). Moreover, *p*-coumaric acid and ferulic acid metabolism were recently engineered in *R. eutropha* by constructing an exogenous, CoA-dependent non-β-oxidation pathway demonstrating the co-utilization of aromatic compounds derived from lignocellulose by the engineered strain (Weng et al. 2023).

Molasses is a by-product from the sugar refining industry that consists mostly of sucrose. By expressing the *Mannheimia succiniciproducens sacC* gene encoding β-fructofuranosidase, that hydrolyses sucrose extracellularly, the substrate spectrum of a glucose-utilizing *R. eutropha* mutant strain, could be expanded to include sucrose (Jo et al. 2021; Park et al. 2015). To enable uptake of sucrose disaccharide in *R. eutropha* H16, the sucrose permease (*cscB)* and sucrose hydrolase (*cscA)* of *E. coli* strain W were heterologously expressed (Arikawa et al. 2017). Nevertheless, cell yields reported on molasses are still limited, probably due to the organic and inorganic inhibitors present in this side-stream that result in inhibited cell growth (Jo et al. 2021). To tackle this, ALE or further genetic engineering should be applied in future to enhance growth of *R. eutropha* on crude molasses.

Starch is one of the most abundant carbohydrates contained in plants (potatoes, wheat, corn, rice, etc.) and consequently often appears in food wastes*. R. eutropha* lacks genes coding for putative amylase or glucoamylase enzymes that could hydrolyze this polysaccharide. Growth of an engineered *R. eutropha* strain on starch as the sole carbon source was demonstrated for the first time recently by expressing two extracellular amylases, the glucodextranase (G1d) from *Arthrobacter globiformis* and the α-amylase (AmyZ) from *Zunongwangia profunda* (Brojanigo et al. 2022). The engineered strain demonstrated high-efficiency enzymatic hydrolysis, which is promising for valorization of starchy waste streams.

Whey is a by-product of the dairy industry with an annual production of over 200 million tons in 2023 (Mukherjee et al. 2023). Upcycling the lactose of this side-stream into value-added compounds using *R. eutropha* necessitates metabolic engineering, as the strain is unable to cleave this disaccharide. Cleavage of lactose can be conferred to a glucose-utilizing strain by heterologous expression of *lacZ* (β-galactosidase), *lacY* (transporter), *lacI* (repressor), and *lacO* (operator) from *E. coli* (Povolo et al. 2010; Pries et al. 1990). Nevertheless, only low cell densities were reported during growth of the engineered strain on lactose with yet great optimization potential.

#### Optimized glycerol utilization

As a main by-product of the biodiesel industry, glycerol is an attractive, cheap feedstock for biobased product synthesis. Glycerol can enter the cytoplasm of *R. eutropha* cells through facilitated diffusion, probably mediated by the glycerol uptake facilitator protein GlpF (H16_A3690) and is then channelled into the ED pathway by the necessary kinases and dehydrogenases (Kaddor & Steinbüchel 2011). *R. eutropha* H16 has been shown to express glycerol metabolic genes when grown on triacylglycerols as the sole carbon source (Brigham et al. 2010). However, low growth rates are exhibited on glycerol, probably due to low uptake rates, insufficient glycerol kinase activity and, additionally, due to high activity of hydrogenases overproducing reactive oxygen species, elevated oxidative stress. Several attempts were undertaken to optimize glycerol utilization in *R. eutropha*. Fukui and coworkers significantly increased assimilation of glycerol by introducing the aquaglyceroporin (*glpF*) and glycerol kinase (*glpK*) genes from *E. coli*, showing that a slight increase in glycerol kinase activity by the heterologous *glpK* imparts a strong effect, nearly quadrupling the growth rate on this substrate (Fukui et al. 2014). Recently, a doubling of the growth rates was achieved by also co-expressing a dehydrogenase (*glpD)* from *E. coli*, as this resulted in accelerated glycerol-3-phosphate conversion to dihydroxyacetone phosphate as compared to that of the native dehydrogenase (Strittmatter et al. 2022). Regarding PHA accumulation on glycerol, it was observed that unspecific incorporation of glycerol by the PHA synthase PhaC can lead to polymer chain termination during prolongated cultivations lowering final molecular weights (Tanadchangsaeng & Yu 2012).

#### Towards the utilization of waste gases: C1 compounds

*R. eutropha* is a well-known facultative chemolithotroph able to utilize gas-phase carbon sources, such as CO_2_ and formate. Syngas is a gaseous by-product of coal-fired power plants, petroleum refineries, and steel mills, which is mainly composed of CO, CO_2_, and H_2_. Syngas holds great potential as feedstock for microbial fermentation as it can support carbon–neutral biomanufacturing reducing GHG emissions (Gutschmann et al. 2022).

*R. eutropha* assimilates CO_2_ via the Calvin–Benson–Bassham (CBB) cycle encoded by two *cbb* operons with the key enzyme RuBisCO responsible for the fixation of CO_2_, while three distinct oxygen-tolerant [NiFe]-hydrogenases deliver energy via H_2_ oxidation (Bowien & Kusian 2002). The pathway operates with a high ATP demand, and RuBisCO exhibits a low catalytic rate, as well as a wasteful side activity (oxygenation) that needs to be remedied through the photorespiration pathway, further increasing the CBB cycle’s expenditure of cellular resources (Della Valle et al. 2024). Several attempts have recently been made to improve autotrophic growth of *R. eutropha*. As only low oxygen concentrations are considered safe for syngas fermentations to avoid explosion risks, a transcriptome-aided engineering strategy was undertaken by Tang et al. by heterologously expressing *vhb* (*Vitreoscilla* hemoglobin) to promote oxygen diffusion into the cell and enhance ATP levels favoring aerobic metabolism under oxygen-limiting conditions (Tang et al. 2020). Further, expression of a heterologous cyanobacterial RuBisCO while overexpressing the endogenous GroES/EL chaperones necessary for correct folding of the enzyme and both soluble and membrane-bound hydrogenases (SH and MBH) led to an increase in growth over 90 % in gas fermentation with 7:1:1 ratio of H_2_, CO_2_, and O_2_ (Li et al. 2020). *β*-carbonic anhydrases (encoded by *can*, *can2*, *caa*, and *cag* genes) catalyze the interconversion between carbon dioxide and bicarbonate and are indispensable for the propagation of *R. eutropha* on atmospheric CO_2_ (Gai et al. 2014). It was shown that tailoring the *can* gene dosage increased cell growth and product formation of the strain (Thorbecke et al. 2021). Recently, another promising strategy to improve carbon fixation was reported by overexpressing the main transcriptional regulator of the *cbb* operon, CbbR, combined with the overexpression of global transcription factor RegA, which resulted in overall enhanced transcription levels of the autotrophic operons (Kim et al. 2022).

*R. eutropha* growth and viability is insensitive to CO in the ratio of conventional syngas but cannot use CO as a carbon source without additional engineering, although it contains certain putative genes in its chromosome that potentially encode carbon monoxide dehydrogenases (CODHs) (Heinrich et al. 2018; Volova et al. 2002; Volova & Voinov 2004). By heterologously expressing the CODH operon from *Oligotropha carboxidovorans*, a recombinant strain could grow on CO as the sole carbon source although supply of H_2_ would be necessary as electrons generated from CO utilization were not directed to the respiratory chain (Heinrich et al. 2018). A recent study used ALE to increase tolerance of *R. eutropha* to high CO concentrations above 50 %, through subculturing on a syngas-like mixture with increasing CO concentrations. Evolved strains showed a mutation on the terminal respiratory cytochrome *bd* ubiquinol oxidase when grown heterotrophically and on the soluble [NiFe]-hydrogenase when grown autotrophically (Wickham-Smith et al. 2023).

Formate can be produced from CO_2_ and renewable electricity, making it a promising feedstock for microbial upgrading. *R. eutropha* has the ability to naturally grow on formate as it is oxidized by two formate dehydrogenases to CO_2_ and fixed in the CBB cycle. One recent study used metabolic engineering in combination with ALE to establish formate metabolism via a synthetic reductive glycine pathway and demonstrated similar yields to the natural pathway (Claassens et al. 2020). Further, another group used ALE to optimize formate utilization. Genome sequencing and transcriptomic analyses of the evolved strains indicated that deletion of a transcriptional regulator implicated in quorum sensing, PhcA, reduced the expression of several operons, and led to improved growth on formate. Furthermore, deleting large regions present on the extrachromosomal megaplasmid pHG1, in particular two hydrogenase operons and the *cbb* operon, further increased growth rates (Calvey et al. 2023).

### Genome-scale metabolic modeling

Genome-scale metabolic models (GSMMs) are mathematical representations encompassing all known biochemical reactions in an organism, each characterized by a rate and a stoichiometry which allow the prediction of the effects of feedstock, environmental conditions, or genetic modifications on the overall metabolism and product formation of a given strain. The advent of whole-genome sequencing and high-throughput data spurred extensive efforts to establish comprehensive and well-curated GSMMs for *R. eutropha* for use in exploring strategies to improve product yields, identifying metabolic bottlenecks, and characterizing the biological mechanisms that regulate process dynamics (Morlino et al. 2023).

The most widely employed mathematical method for studying GSMMs is flux balance analysis (FBA). FBA generates a system of linear equations based on metabolic reactions and their constraints, allowing the identification of optimal flux distributions through linear programming. Despite their utility, the construction and maintenance of reliable GSMMs demand extensive manual curation and validation with multi-omic data. The first *R. eutropha* model, RehMBEL1391, was published in 2011 (Park et al. 2011) and was built on reactions annotated in the Kyoto Encyclopedia of Genes and Genomes database (KEGG; https://www.genome.jp/kegg-bin/show_organism?org=reh), which were based on the published genome sequence data (Pohlmann et al. 2006). Unfortunately, this model was not computer-readable and did not use specific metabolite and reaction IDs, hindering efforts to automatically update it. Recently, the model was updated by addition of annotations and ID mapping for genes, reactions, and metabolites (Jahn et al. 2021). By validating the model with protein quantification data, the authors suggested that, under controlled fermentation conditions, over 40% of the proteome of *R. eutropha* is not utilized, leaving great potential for genetic reduction and protein resource reallocation. Pearcy and coworkers published an advanced model in 2022, iCN1361, which was directly constructed from the BioCyc Pathway Genome DataBase (PGDB) in an effort to improve its readability and reusability, visualization, and update with new omics data (Pearcy et al. 2022). With 1292 reactions (98 of which are transporters) and 1265 metabolites, the model fully obeys the law of conservation of mass for carbon, nitrogen, sulfur, oxygen, phosphate, and hydrogen (including protons), and is also free from erroneous energy-generating cycles. Apart from predicting *R. eutropha* growth phenotypes for a variety of feedstocks, the model was able to accurately predict gene knockout phenotypes which was verified by a Transposon-directed Insertion site Sequencing (TraDIS) approach for genome-wide essentiality screening (Pearcy et al. 2022).

It is important to recognize that GSMMs provide a simplified representation of metabolism, without consideration of enzyme kinetics, thermodynamic feasibility, and gene expression. Consequently, they may occasionally yield solutions that are not feasible in vivo. Despite these limitations, GSMMs remain invaluable computational tools offering useful insights for designing metabolic engineering strategies and saving time and resources by predicting optimal process parameters. Despite the potential of GSMMs, their application to improve culturing conditions or optimize genetic manipulation in *R. eutropha* remains largely unexplored, likely due to the only recent development of advanced models. In other organisms, GSMMs have already found their applications in nearly every aspect of biological research, including elucidating metabolic pathways, predicting gene essentiality, industrial applications, and drug discovery (Passi et al. 2022).

### Minimal cells

The ideal microbial chassis should be easily reprogrammable to efficiently utilize diverse carbon sources to manufacture value-added compounds on demand in a predictable and robust manner. However, living organisms are inherently complex, exhibiting variable metabolic states with difficult-to-predict and intricately controlled gene expression, and native gene networks often interfere with heterologous pathways, which may also be disrupted by defensive transposable elements universally present in most organisms (Fan et al. 2020). Therefore, reducing genome complexity by minimizing unnecessary DNA sequences and creating simpler life forms that express synthetic genetic circuits without host interference is a key target of modern synthetic biology (Moya et al. 2009). So-called minimal cells should retain all essential genes involved in basic housekeeping functions, alongside a limited set of metabolic processes needed to survive in a nutrient-rich and stable environment while enabling the development of smart biomanufacturing systems (Della Valle et al. 2024). There are two strategies for genome size reduction: top-down, involving sequential removal of unnecessary genome sequences, and bottom-up, with synthetic genomes created from scratch (Chen et al. 2023). Involving the former, deletion of the megaplasmid pHG1 of *R. eutropha* led to 24 % faster growth rates on formate (Calvey et al. 2023) providing evidence for the synergistic combination of ALE and global transposon mutagenesis for predicting non-essential genes. Regarding the latter, recently a groundbreaking advancement was reported as chromosome-free cells (simple cells (SimCells) or non-replicating minimal cells) were generated from *R. eutropha* by expressing an intron-encoded endonuclease, I-Ceul, that recognizes and cleaves a conserved region of the 23S rRNA present in most bacterial genomes, degrading the genome while leaving heterologous plasmids without such recognition sites unaffected (Fan et al. 2020). The cells successfully expressed a heterologous glycolysis pathway and maintained their structural integrity and enzymatic activity for a few days with cellular ATP and NDPH levels depleted only at day 10, therefore being robust enough to remain functional for most biomanufacturing applications. SimCells are a step forward in addressing the need for a robust and reprogrammable microbial chassis as they avoid the possibility of genetic drift and evolvability due to the inability to replicate, which also alleviates some of the biosafety concerns associated with genetically modified microorganisms. The chromosome-free SimCells have the potential to revolutionize synthetic biology by enabling the development of smart biomanufacturing systems.

### Application and products

*R. eutropha* has demonstrated remarkable efficiency in producing various PHAs from diverse waste carbon sources including naturally occurring PHB (Loan et al. 2022; Nangle et al. 2020), as well as P(HB-*co*-HV) (Jawed et al. 2022), and engineered variants such as P(HB-*co*-HHx) (Gutschmann, et al. 2023a, 2023b; Riedel et al. 2023), P(HB-*co*-LA) (Jo et al. 2021), and P(HB-*co*-H4MV-*co*-H2MP) (Wang et al. 2024a). Among these, P(HB-*co*-HHx) stands out for its potential to substitute conventional plastics due to its ability to offer a wide range of properties through the manipulation of its monomer composition (Thiele et al. 2024a, b). Recent studies showed that the HHx monomer content derived from palm kernel oil can be increased up to 36 mol% by deleting β-ketothiolase genes involved in the β-oxidation of C6 substrates while overexpressing the (*R*)-specific enoyl-CoA hydratase PhaJ, thus maximizing the flow to HHx precursors (Arikawa & Sato 2022); additionally, P(HB-*co*-HHx) with up to 70 mol% HHx was produced from fructose by rewiring the formation of HHx precursors through the fatty acid de novo synthesis (Park et al. 2024). Furthermore, the molecular weight of PHA polymers also strongly influences their processability and it was shown that by knocking out depolymerases PhaZ1, PhaZ2, and PhaZ6, *R. eutropha* can produce ultra-high-molecular-weight PHA that is not degraded under carbon starvation (Arikawa et al. 2016). In *R. eutropha*, PHA accumulation is induced under unbalanced growth conditions, such as inorganic nutrient limitation (P, N, O_2_), which impairs the synthesis of essential cellular components, leading to the intracellular accumulation of acetyl-CoA and increased NAD(P)H levels, which allosterically inhibit the enzymes of the TCA cycle. The main PHA biosynthetic pathway consumes NADPH as a cofactor for the conversion of acetoacetyl-CoA to 3-hydroxybutyryl-CoA mediated by PhaB, and thus strategies aimed at increasing the levels of this reducing equivalent are of particular interest for enhancing PHA productivities (Choi et al. 2003; Lee et al. 2003). Regarding the recovery of PHAs, recently *R. eutropha* was engineered for increased susceptibility to osmolysis by increasing halotolerance by ALE and knocking out the large-conductance mechanosensitive channel (*mscL*) gene which improved osmolytic efficiency upon osmotic downshock by 90 % (Adams et al. 2023).

Apart from being the model platform for the production of PHA bioplastics, *R. eutropha* has been pointed out as a game-changer for mitigating global warming due to its potential as a biofuel producer (Brigham 2019; Sohn et al. 2021) including alcohols like isopropanol (Bommareddy et al. 2020; Boy et al. 2023; Liu et al. 2016; Garrigues et al. 2020; Grousseau et al. 2014b; Marc et al. 2017; Subagyo et al. 2021; Torella et al. 2015), isobutanol (Bernardi et al. 2016; Black et al. 2018; Liu et al. 2016; Lu et al. 2012) and bioethanol (Lee et al. 2016), fatty acids (Li et al. 2019; Chen et al. 2015), methyl ketones (Müller et al. 2013), alkanes and alkenes (Bi et al. 2013; Crépin et al. 2018), and even terpenoids including isoprene (C5) (Lee et al. 2019) and β-farnesene (C15) (Milker & Holtmann 2021). The production of other terpenoids including α-humulene (C15) (Krieg et al. 2018; Langsdorf et al. 2022; Milker et al. 2021; Sydow et al. 2023), a potential drug to treat cancer, and lycopene (C40) (Wu et al. 2022), used in the cosmetic industry, has also been reported recently. As a common rule for all engineering efforts, apart from establishing efficient heterologous biosynthetic pathways, product yields are generally enhanced by the blocking of competing metabolic pathways while also increasing the tolerance to potentially toxic products. Regarding the carbon feedstock, both heterotrophic and autotrophic production of valuable biofuels and platform chemicals can be achieved by engineered *R. eutropha*. Although the latter shows significantly lower yields, it has the primary advantage of valorizing CO_2_, thereby reducing GHG emissions. Microbial electrolysis systems (MES), in which redox equivalents (formate, H_2_, and electrons) needed by microorganisms to upgrade CO_2_ into value-added compounds are provided via electrochemical reactions fuelled by renewable energy, are considered promising in this context. Nevertheless, challenges arise in the coupling of *R. eutropha* with the inorganic system as reactive oxygen species (ROS), with hydrogen peroxide being a major component, are generated.

Recent advancements have also demonstrated the versatility of the organism for efficient bioconversion of waste streams into other novel products including platform chemicals, dietary supplements, and vitamins (Table 2).

**Table 2:** Product portfolio of *R. eutropha*

Product	Carbon source	Strain	Yield and scale	Reference
1,3-butanediol	CO_2_	H16 *ΔphaC1 ΔsucCD*,* P[bld yqhDEc dra pdc phaAB]*	2.97 g L^−1^ Shake flask	(Gascoyne et al. 2021)
Acetoin	CO_2_	H16 Δ*phaC1* Δ*phaC2* Δ*acoABC*, *P*_*PhaC1*_-*alsSD*	3.9 g L^−1^ Batch lab-bioreactor	(Windhorst & Gescher 2019)
Alka(e)ne	Fructose	H16 *ΔphaCAB*,* P*_*araBAD*_*-ado-aar*	1.5 g L^−1^ Fed-batch lab-bioreactor	(Crépin et al. 2018)
Cyanophycin	Fructose	H16PHB-4 *Δeda*,* P*_*lacPOZ*_*-cphA-eda*	31.3 g L^−1^ Fed-batch lab-bioreactor	(Lin et al. 2012)
Fatty acids	CO_2_	H16 *ΔphaC1*, P_araBAD_-*acc-LTes*, P_araBAD_-*fas-acpS*	60.64 mg L^−1^ Continuous lab-bioreactor	(Li et al. 2019)
Isobutanol	Fructose	H16 Δ*phaCAB* ΔilvE, ΔbkdAB ΔaceE, P_*lac*_-*ilvBHCD*-*kivd*	14 g L^−1^ Shake flask	(Lu et al. 2012)
Isopropanol	Fructose	H16 *ΔphaC1 ΔphaB1B2B3*, ParaBAD-phaA-ctfAB-adc*-adh*;* P*_*lac*_*-groESL*	15.1 g L^−1^ Fed-batch lab-bioreactor	(Boy et al. 2023)
Lipochitooligosacharide	CO_2_	H16, *P*_*araBAD*_*-nodABC*	1.4 mg L^−1^ Shake flask	(Nangle et al. 2020)
L-valine	CO_2_	H16 *ΔH16_A0006 ΔphaCAB ΔnagR nagE*_*G793C*_ P_*phaC1*_-*ygaZH* P_*phaC1*_-*ilvBHEC*	319 mg L^−1^ Shake flask	(Wang et al. 2024b)
Lycopene	CO_2_	H16 *ΔH16_A0006 ΔH16_0008-9*,* P*_*lac*_*-CrtEBI2*	1.73 mg L^−1^ MES	(Wu et al. 2022)
Mannitol	CO_2_	H16 *ΔphaCAB,* P_*araC*_-*araC*, P_*araBAD*_-*mtlD/ m1p*a	3.9 g L^−1^ Batch lab-bioreactor	(Hanko et al. 2022)
Methyl-ketones	CO_2_	H16 *ΔphaCAB Δ(*A0459-0464, A1526-1531), P_*araBAD*_- *tesA*-*fadBMlut_11700*-*fadM*	180 mg L^−1^ Batch lab-bioreactor	(Müller et al. 2013)
Myo-inositol	CO_2_	H16 *ΔnagE ΔnagR ΔH16_A0006*,* P*_*phaC1*_*-imp-ips*	1.05 g L^−1^ Shake flask	(Wang, et al. 2023c)
*N*-acetylglucosamine	CO_2_	H16 *ΔphaCAB ΔnagFECAB ΔH16_A0006*, *P*_*PhaC1*_-Cegna1*	75.3 mg L^−1^ Shake flask	(Wang et al. 2023b)
PHB	CO_2_	DSM531	61.9 g L^−1^ Batch lab-bioreactor	(Tanaka et al. 1995)
P(HB-*co*-HV)	Butyrate + propionate	DSM545	58 g L^−1^ Fed-batch lab-bioreactor	(Grousseau, et al. 2014a)
P(HB-*co*-HHx)	Palm kernel oil	H16 *ΔbktB ΔH16_A1528 ΔphaC1 ΔphaZ1Z2Z6*,* P*_*trc*_*- phaJ4b, P*_*trp*_*-phaC*_*AC*_	196 g L^−1^ Fed-batch lab-bioreactor	(Arikawa & Sato 2022)
	Fructose + canola oil	H16 *ΔphaC1*,* P*_*PhaC1*_*-phaC2*_*Ra*_*-phaA-phaJ1*_*Pa*_	107 g L^−1^ Repeated fed-batch lab-bioreactor	(Santolin et al. 2021)
			71 g L^−1^ Fed-batch pilot-scale	(Thiele et al. 2024a)
α-humulene	CO_2_	H16PHB − 4, P_*rhaBAD*_-*zssI*-*erg20*-*hmgSfni*- *hmgR*-*mvaK*-*mvaDmvaK2*	146 mg L^−1^ Batch lab-bioreactor	(Sydow et al. 2023)

#### “Novel” applications of *R. eutropha*

##### Mannitol

D-Mannitol is a sugar alcohol, naturally present in many plant species, that is widely marketed as a low-caloric sweetener and utilized in medicine as a diuretic agent and in the pharmaceutical industry in the formulation of tablets (Song & Vieille 2009). Recently, a groundbreaking achievement was made with the de novo biosynthesis of this industrially significant sugar alcohol in *R. eutropha*, resulting in 3.9 g L^−1^ mannitol production using CO_2_ as the substrate (Hanko et al. 2022). For this purpose, a mannitol biosensor was developed to screen for the best combination of heterologous mannitol biosynthetic genes while identifying optimal gene expression conditions. The biosensor harbored combinations of mannitol 1-phosphate dehydrogenase (MtlD) and mannitol 1-phosphate phosphatase (M1P), derived from different organisms, under control of AraC/P_*araC*_. Upon addition of arabinose, MtlD/M1P catalyzed the conversion of fructose-6-phosphate into mannitol accompanied by the expression of red fluorescence protein (*rfp*) through the activation of P_mtlE_ by its corresponding mannitol-responsive transcriptional regulator MtlR. The authors demonstrated mannitol production via highly efficient glyceraldehyde-3-phosphate flow of the CBB cycle into fructose-6-phosphate highlighting the remarkable potential of *R. eutropha* in converting CO_2_ into valuable sugar derivatives.

##### NAG

*N*-acetylglucosamine (NAG) is a bioactive amino sugar widely used as a dietary supplement due to its beneficial effects against arthritis, neurological disorders, and inflammatory health ailments (Ahuja et al. 2021). Recently, a biosynthetic pathway for the conversion of CO_2_ into NAG was established in *R. eutropha* providing one further feasible approach to utilize this bacterial chassis in the production of bioactive chemicals (Wang et al. 2023b). To this end, the genes coding for the NAG porin (*nagC*), PTS system (*nagF* and *nagE*), deacetylase (*nagA*), and deaminase (*nagB*) were knocked out from the genome of *R. eutropha* to prevent the import and catabolism of this compound. Subsequently, NAG biosynthesis was established by heterologous overexpression of *gna1* from *Caenorhabditis elegans*, coding for an *N*-acetylglucosamine-6-phosphate *N*-acetyltransferase that catalyzes the formation of *N*-acetylglucosamine-6-phosphate (GlcNAc-6-P) from glucosamine-6-phosphate previously synthetized from fructose-6-phosphate by a glucosamine-6-phosphate synthase (GlmS) naturally encoded by *R. eutropha*. GlcNAc-6-P can be further dephosphorylated by native haloacid dehalogenase (HAD) phosphatases (CbbY2 and CbbYp) leading to NAG. The authors showed increased NAG yields by disrupting PHB biosynthesis and ED pathways yielding up to 75.3 mg/L NAG during autotrophic growth.

##### Acetoin

Another novel platform chemical that has recently been produced by engineered *R. eutropha* is acetoin, used as precursor for polymer or fuel production or as flavoring compound with a butter-like taste (Xiao & Lu 2014). For the autotrophic production of acetoin, the *acoABC* operon of *R. eutropha* was first knocked out to prevent consumption of the compound, and then codon-optimized versions of the *B. subtills alsS* (acetolactate synthase) and *alsD* (acetolactate decarboxylase) genes responsible for the synthesis of acetoin from pyruvate under control of P_*PhaC1*_ promoter were engineered (Windhorst & Gescher 2019). By deleting *phaC1* and *phaC2* and directing carbon flux to product formation, the strain achieved acetoin production with 100% efficiency from CO_2_. Recently, the same strain was shown to produce acetoin also from propionate as sole carbon source and under mixotrophic growth, although both with lower carbon efficiencies than using solely CO_2_ (Härrer et al. 2021).

##### Lipochitooligosacharide (LCO)

In a further effort to decouple the bioproduction of chemicals from plant-based feedstocks, the autotrophic synthesis of a lipochitooligosacharide (LPO) plant growth enhancer was recently realized by heterologously expressing NodC (an N-acetylglucosaminyltransferase that builds the backbone using glucose as a precursor), NodB (a deacetylase that acts on the non-reducing end), and NodA (an acetyltransferase that attaches a fatty acid) from *Bradyrhizobium japonicum* under control of P_*BAD*_ in *R. eutropha* (Nangle et al. 2020). The production of the synthetic fertilizer, which showed equivalent yields to those achieved by its native source, was produced in an integral approach using engineered *R. eutropha* for the coupled autotrophic production of LCO, sucrose and various PHAs.

##### (R)-1,3-butanediol

1,3-butanediol (1,3-BDO) is an important platform chemical used in the production of synthetic rubber, polyester resins, and insecticides and as a key intermediate in the production of β-lactam antibiotics (Duan et al. 2016). *R. eutropha* was recently engineered for the efficient production of 1,3-BDO from CO_2_ showing once more the potential of redirecting acetyl-CoA to the production of novel value-added compounds (Gascoyne et al. 2021). In the study, two alternative heterologous (*R*)-1,3-BDO biosynthetic pathways, based on utilization of either (*R*)-3HBCoA or pyruvate as precursors, were engineered. In the first pathway, (*R*)-3HBCoA was converted by AdhE2 (aldehyde-alcohol dehydrogenase) from *C. acetobutylicum* or a combination of codon-optimized Bld (butanal dehydrogenase) from *C. saccharoperbutylacetonicum* and *E. coli* YqhD (aldehyde reductase) into (*R*)-1,3-BDO; in the second pathway, heterologous expression of *pdc* (pyruvate decarboxylase*)* from *Z. mobilis*, *dra* (deoxyribose-5-phosphate aldolase) and *yqhD* from *E. coli* directed flux from pyruvate into (*R*)-1,3-BDO. Combination of both pathways while abolishing PHA synthesis and reducing the flux through the tricarboxylic acid cycle enabled to produce over 2.97 g L^−1^ of (*R*)-1,3-BDO via autotrophic fermentation on CO_2_.

##### Myo-inositol

*Myo*-inositol is a cyclitol B-group vitamin with reported benefits for the treatment of depression, Alzheimer’s disease, panic disorder, and fatty liver (Li et al. 2022). Production of *myo*-inositol from CO_2_ was reported recently using engineered *R. eutropha* with a yield of > 1 g L^−1^ (Wang, et al. 2023c). The authors engineered a glucose-utilizing strain with a *myo*-inositol-3-phosphate synthase (IPS) from either *Trypanosoma brucei* or *S. cerevisiae* and an inositol monophosphatase (IMP) from *E. coli*, which enabled synthesis of *myo*-inositol from glucose, glycerol, and CO_2_ through glucose-6-phosphate and showed optimized yields by inactivating the ED and PHA synthesis pathways.

##### Cyanophycin

Cyanophycin, also known as cyanophycin granule polypeptide (CGP), is a non-ribosomally synthetized polypeptide composed of a polyaspartic backbone and arginine-side chains that are of biotechnological interest due to its application in the chemical and drug industries (Simon & Weathers 1976). Although *R. eutropha* harbors two cyanophycin synthetase homologs in its genome, no cyanophycin production has been observed in the wild-type strain (Adames et al. 2013). The maximum cell-density production of cyanophycin in recombinant *R. eutropha* was reported using a PHB^−^ mutant harboring a cyanophycin synthetase from *Synechocystis* sp. and ensuring plasmid stability with a KDPG aldolase gene-dependent addiction system (Lin et al. 2012).

## Outlook

*R. eutropha* is a synthetic biology chassis organism that is currently being explored for a variety of applications. Most of these applications take advantage of two particular aspects of the bacterium: its metabolic versatility and its genetic manipulability. Collection and characterization of effective genetic regulatory elements like promoters and RBSs (Alagesan et al. 2018) provide the basis for building a toolbox of standardized parts for *R. eutropha*. As summarized above, in addition to the “classic” applications of *R. eutropha* (i.e., PHA, biofuels), new applications for this versatile organism are being explored.

The continued exploration of the synthetic biology of *R. eutropha* holds significant promise. We can consider it a given that the versatile metabolism of *R. eutropha* will continue to be explored and rewired to produce novel and useful bioproducts. It is generally accepted in biomanufacturing that the expression of a genetic device in a heterologous host to produce a novel product or impart novel capability results in metabolic burden on that host. Burden is generally a result of a competition of resources between the microbial chassis and the synthetic construct (Boo et al. 2019). We can address the burden in polymer productions by formulating the cultivation conditions so that *R. eutropha* synthesizes PHA only under controlled nutrient limitation. We are able to control PHA production cultivations in this way because of the natural role of the polymer in cells. However, the bioengineer cannot necessarily exploit these facets of *R. eutropha* physiology when the host harbors a synthetic construct to perform a novel task. To maximize the production of novel biochemicals, devices must be designed with the alleviation of metabolic burden in mind. Development of synthetic biology tools and techniques for *R. eutropha* can allow for more user-friendly design and construction of production strains that have growth and production decoupled.

To continue to inform *R. eutropha* production strain construction, the genome-scale metabolic models can be curated to include the large amount of gene expression, proteomic, metabolomic, and physiological data available today. These models can also help decode roles of previously uncharacterized or function-unknown genes and gene products. From studies such as these, a roadmap to the construction of a *R. eutropha*-based minimal genome cell can be designed.

Since its discovery and characterization over 50 years ago, *R. eutropha* has become a model organism for PHA homeostasis, as well as an industrially relevant organism. From many of the studies summarized here, it is clear that a synthetic biology toolkit is being established for *R. eutropha*, which will enable us to drive this microbial chassis into the future.

## Data Availability

Not applicable.
